# Association of legumes and nuts consumption with metabolic health status in Iranian overweight and obese adolescents

**DOI:** 10.1038/s41598-023-32961-2

**Published:** 2023-04-08

**Authors:** Houri Heshmatipour, Zahra Hajhashemy, Saeideh Mirzaei, Ali Asadi, Masoumeh Akhlaghi, Parvane Saneei

**Affiliations:** 1 Department of Community Nutrition, School of Nutrition and Food Science, Nutrition and Food Security Research Center, Students’ Research Committee, Isfahan University of Medical Sciences, Isfahan, Iran; 2 Department of Community Nutrition, School of Nutrition and Food Science, Shiraz University of Medical Sciences, Shiraz, Iran; 3 Department of Exercise Physiology, School of Physical Education and Sport Sciences, University of Tehran, Tehran, Iran; 4 Department of Community Nutrition, School of Nutrition and Food Sciences, Shiraz University of Medical Sciences, Shiraz, Iran; 5 Department of Community Nutrition, School of Nutrition and Food Science, Nutrition and Food Security Research Center, Isfahan University of Medical Sciences, Isfahan, PO Box 81745-151, Iran

**Keywords:** Metabolic disorders, Nutrition disorders

## Abstract

Limited data are available on the association of legumes and nuts consumption with health status in pediatrics. So, we assessed the relation of legumes and nuts intake with metabolic health status in Iranian adolescents. A random sample of overweight/obese adolescents aged 12 to 18 years was included in this cross-sectional study. Dietary intakes were gathered using a validated 147-item food frequency questionnaire (FFQ). We measured metabolic indices including blood pressure, lipid profile, glycemic and anthropometrics indices. Two strategies were used for classification of adolescents to metabolically healthy obese (MHO) or unhealthy obese (MUO): International Diabetes Federation (IDF) and combination of IDF with Homeostasis Model Assessment Insulin Resistance (HOMA-IR). Information on 203 overweight/obese adolescents (50.2% girls and 49.8% boys) with an average age of 13.98 (± 1.61) years and a mean weight of 73.48 (± 11.60) kg/m^2^ was evaluated. Based on the IDF and IDF/HOMA-IR definition, higher consumption of legumes and nuts consumption was related to a 66% and 61% decreased odds of MUO in crude model (OR = 0.34, 95%CI 0.17–0.69), (OR = 0.39, 95%CI 0.19–0.80); but in fully-adjusted model, these relations disappeared. After adjustment for potential cofounders, an inverse association was found between legumes and nuts consumption and odds of hyperglycemia (OR = 0.35, 95%CI 0.16–0.78). Moreover, although inverse significant associations were found between legumes and nuts consumption and odds of MUO in girls and overweight subjects in crude models, these associations disappeared after adjustment for all confounders. After taking potential confounders into account, no significant association was found between consumption of legumes and nuts and MUO in Iranian adolescents. The findings should be affirmed by further prospective studies.

## Introduction

Overweight and obesity prevalence have increased significantly over the past four decades^1^. Based on the Global Burden of Disease (GBD) data, the overweight prevalence in children and obesity in adolescents will be increased to 268 and 124 million by 2025, in the world^1^. It was estimated that 4 million adolescents with excess weight will live in Iran by 2025^2^. Obesity in childhood has an increased burden on healthcare systems^3^, because it is related to complications like insulin resistance, dyslipidemia, impaired glucose metabolism, high blood pressure (BP), and some cancers^4–6^. Previous evidences documented that children with obesity may still be obese in adulthood^4^ and had an increased risk of cardiac events^7^ and all-cause mortality in adulthood^8^. Individuals with obesity have one of two phenotypes of metabolically healthy overweight/obese (MHO) or metabolically unhealthy overweight/obese (MUO)^6,9^. MHO is a dynamic state before MUO and MUO is a progressive state^6,10^. Overweight/obese subjects with at least 2 metabolic disorders (including, hyperglycemia, hypertension, dyslipidemia (low serum HDL-c and/or hypertriglyceridemia), and insulin resistance) are classified as MUO. On the other hand, overweight/obese subjects with less than 2 above-mentioned disorders are defined as MHO^11,12^.

Several risk factors are involved in the etiology of MUO such as age, genetic predispositions, lifestyle factors and their interactions^10,13^. A healthy diet has a beneficial effect on metabolic profile^10^; such that, a higher Dietary Approaches to Stop Hypertension (DASH) score was adversely related to MUO odds in obese children^9^. Additionally, adherence to a Mediterranean-style diet improved glucose, body mass index (BMI), total cholesterol (TC), triglycerides (TG), high density lipoprotein cholesterol (HDL-c), and low density lipoprotein cholesterol (LDL-c), in Mexican obese children and adolescents^14^. Furthermore, a cohort study indicated that nuts consumption decreased the values of TG, waist circumference, systolic blood pressure (SBP), BMI and weight^15^. Several legumes, such as soy, have functional properties that have favorable effects on cholesterol, TG, glucose metabolism, blood pressure and inflammation^16^. A systematic review and meta-analysis of prospective studies demonstrated that legumes consumption reduce the risk of overweight/obesity and weight gain^17^. To our knowledge, the association between food groups and MUO or MHO has less been studied, and most former studies were conducted in European and USA populations^18^. So, the current study was conducted to assess the relation of legumes and nuts consumption with metabolic health status in Iranian overweight/obese adolescents.

## Methods and materials

### Participants and study design

Overall 203 overweight/obese adolescents (101 boys and 102 girls) aged between 12 to 18 years were included in this cross-sectional study. Using a stratified, multi-stage cluster sampling method, subjects were selected from 16 schools of 5 different districts of Isfahan, Iran in 2020. By this method, students with different socio-economic statuses were considered for the study. Taking a prevalence of MUO in overweight and obese Iranian adolescents (60%)^19,20^, precision (d) of 7%, 0.95 confidence interval (CI), 0.05 type I error and 80% power into account, at least 188 subjects were required to be included in the current analysis. Only overweight and obese adolescents (based on growth curve age-sex specific BMI percentiles^21^) were selected for the study. We did not include subjects that followed a weight-loss diet or had an endocrine disorder or were taking supplements or medications that might influence their metabolic status. All subjects and their parents signed a written informed consent. The local Ethics Committee of Isfahan University of Medical Sciences confirmed the study protocol.

### Assessment of dietary intake

Dietary intakes were gathered using a validated 147-item food frequency questionnaire (FFQ)^22^. Previous studies demonstrated that this FFQ could be a valid and reliable tool for assessing food and nutrient intake among Iranian adolescents^23,24^. To complete the FFQs, a trained dietitian asked the adolescents to report the frequency (based on daily, weekly, monthly) and quantity (based on common portion sizes) of their food intakes in the preceding year. By using household measures^25^, the portion size of food intake was converted to g/day. Finally, Nutritionist IV software was used to estimate the overall intake of energy and nutrients per day. Consumption of nuts was defined as intake of tree nuts (including almond, walnut, hazelnuts, peanuts, seeds, and pistachio) and consumption of legumes was defined as intake of beans, peas, lentil, split peas, mung beans and soy.

### Assessments of anthropometric indices and cardiometabolic risk factors

A trained dietitian evaluated all anthropometric indices. Height of participants was assessed with a stadiometer (to the nearest 0.1 cm) without shoes and with relaxed shoulders. Weight was measured with a calibrated electronic scale (Seca Instruments, Germany) (to the nearest 100 g) with minimum clothes and without shoes. After that, BMI was computed based on the Quetelet formula (weight(kg))/height^2^) and individuals were categorized as obese (> 95th percentile), overweight (85–95th percentile) and normal (< 85th percentile) based on the sex, and age-specific World Health Organization (WHO) cut-off points for adolescents^21^. Only overweight and obese adolescents were selected for the study. The measurement of SBP and diastolic blood pressure (DBP) was done through the use of a mercury sphygmomanometer with an appropriate cuff for blood pressure assessment in this age group; such that, the width of the cuff was approximately 40% of the arm circumference and the bladder cuff length covered approximately 80% of the arm circumference in adolescents^26,27^. The values of BP were recorded two times for each subject and the average of two measurements was considered as the final value.

Based on the standard protocol for biochemical values, blood collection was conducted in a sitting position and after a 12-h overnight fasting. Based on the enzymatic colorimetric method, fasting blood glucose (FBG) was measured using the glucose oxidase enzyme. After precipitation of the apolipoprotein B-containing lipoproteins, HDL-c values were assessed using phosphotungstic acid. Using the enzymatic colorimetric tests, TG values were measured by glycerol phosphate oxidase. Moreover, the values of serum insulin were assessed through the use of the ELISA kits. In order to estimate insulin resistance (IR), Homeostasis Model Assessment Insulin Resistance (HOMA-IR) was computed using the following formula^28^:

$${\text{HOMA}} - {\text{IR}}\, = \,[\left( {{\text{fasting insulin }}\left( {{\text{mU}}/{\text{L}}} \right)\, \times \,{\text{FBG }}\left( {{\text{mmol}}/{\text{L}}} \right)} \right] \, /{22}.{5}.$$

### Assessment of metabolic status

Two strategies were applied to classify adolescents into MUO and MHO. According to the international diabetes federation (IDF) criteria^11^, overweight and obese individuals with two or more of these criteria were considered as MUO: increased TG (≥ 150 mg/dL), decreased HDL-c (< 40 mg/dL for the age of < 16 years, and < 50 mg/dL in female adolescents/ < 40 mg/dL in male adolescents for the age of ≥ 16 years), increased fasting blood glucose (≥ 100 mg/dL) and increased blood pressure (≥ 130/85 mmHg); individuals with less than two above-mentioned criteria were considered as MHO. The second strategy (IDF/HOMA-IR) was the combination of the first criteria (IDF) and HOMA-IR score as an indicator of insulin resistance^12^; such that, overweight/obese individuals with two or more above-mentioned metabolic disorders and insulin resistance (HOMA-IR score ≥ 3.16) were defined as MUO and individuals with HOMA-IR < 3.16 were defined as MHO. The cut-off point of 3.16 was determined based on previous studies on adolescents^29,30^.

### Assessment of other variables

Using the valid Physical Activity Questionnaire for Adolescents (PAQ-A), physical activity was assessed. This questionnaire includes 9 items, the first 8 items are about usual physical activities and the last one is about unusual activities in the last week^31^. Each of the first 8 items has a 1–5 score; score 1 shows the lowest level of activity, and score 5 shows the highest level of activity. Scores were calculated and students were categorized as high active (score ≥ 3) and low active (score < 3). Information of age, sex, drug and supplement use history and other confounders were assessed using a valid demographic questionnaire. Using a validated questionnaire^32^, the socioeconomic status (SES) of adolescents was evaluated based on parental education, parental job, number of family members, having car in the family, having computer/laptop, having personal room and number of trips per year.

### Statistical analysis

The normality distribution of quantitative variables was examined using the Kolmogorov–Smirnov test. The mean ± SD/SE and frequency (percentage) were reported for continuous variables and qualitative variables, respectively. Energy-adjusted values of legumes and nuts intake were provided by the use of the residual method. First, a linear regression between legumes and nuts consumption and total energy intake was calculated. Then, energy-adjusted values of legumes and nuts were computed by summing the mean consumption of legumes and nuts with the residual values obtained from the linear regression. Participants were categorized according to energy-adjusted tertiles of legumes and nuts consumption. Using the chi-square test and one-way analysis of variance (ANOVA), the categorical and continuous variables were reported across energy-adjusted tertiles of legumes and nuts consumption. If ANOVAs were significant, post hoc comparisons by Bonferroni correction were performed. Additionally, analysis of covariance (ANCOVA) was used to determine the energy, age, and sex-adjusted dietary intakes of subjects across energy-adjusted tertiles of legumes and nuts consumption. Using binary logistic regression test, we reported crude and multivariable-adjusted odds ratios (ORs) for MUO (based on IDF and IDF/HOMA-IR definitions) across energy-adjusted tertiles of legumes and nuts consumption. In the first model, adjustments were made for energy, sex and age. In the second model, we additionally made adjustments for physical activity and socioeconomic status. Finally, we added BMI to the adjustments, in the third model. The first category of legumes and nuts consumption was considered as the reference category in all models. In order to determine the trends, energy-adjusted tertiles of legumes and nuts consumption were treated as ordinal variables in logistic regression models. Additionally, we performed a stratified analysis based on BMI categories of participants and their sex. We conducted all analyses through the use of SPSS software version 20. We considered P-values < 0.05 as statistically significant.

### Ethical approval and consent to participate

The study procedure was performed according to declaration of Helsinki and STROBE checklist. All subjects and their parents signed a written informed consent. The study protocol was approved by the local Ethics Committee of Isfahan University of Medical Sciences.

## Results

In this study, information on 203 overweight/obese adolescents (50.2% girls and 49.8% boys) with an average age of 13.98 (± 1.61) years and a mean weight of 73.48 (± 11.60) kg was evaluated. General characteristics and cardiometabolic risk factors of study participants in energy-adjusted tertiles of legumes and nuts consumption are provided in Table 1. Adolescents in the third tertile in comparison to persons in the first tertile were younger. Moreover, participants in the second tertile had higher levels of HDL-c, compared to those in the lowest tertile.

**Table 1:** General characteristics and cardiometabolic factors of study participants across energy-adjusted tertiles of legumes and nuts consumption.

Energy-adjusted tertiles of legumes and nuts consumption				
	T1 (n = 67) (< 41.1 g/day)	T2 (n = 68) (41.1–71.1 g/day)	T3 (n = 68) (> 71.7 g/day)	P-value^a^
Sex, n (%)				0.97
Boys	34 (50.7)	34 (50.0)	33 (48.5)	
Girls	33 (49.3)	34 (50.0)	35 (51.5)	
Age (year)	14.39 ± 1.63*	13.76 ± 1.46	13.78 ± 1.67*	0.04
Weight (kg)	76.28 ± 12.05	71.98 ± 10.52	72.22 ± 11.85	0.05
Height (cm)	165.09 ± 8.78	163.50 ± 7.24	162.32 ± 7.62	0.13
BMI (kg/m^a^)	27.87 ± 2.93	26.83 ± 2.66	27.37 ± 3.94	0.17
Physical activity levels, n (%)				0.01
Low active	43 (64.2)	36 (52.9)	23 (33.8)	
Active	24 (35.8)	32 (47.1)	45 (66.2)	
Socioeconomic status^b^, n (%)				0.16
Low	26 (38.8)	19 (27.9)	14 (20.6)	
Medium	27 (40.3)	32 (47.1)	31 (45.6)	
High	14 (20.9)	17 (25.0)	23 (33.8)	
Systolic blood pressure (mmHg)	115.96 ± 12.00	111.97 ± 21.33	110.24 ± 20.02	0.18
Diastolic blood pressure (mmHg)	75.41 ± 10.25	72.19 ± 13.30	72.91 ± 10.19	0.23
Fasting blood glucose level (mg/dL)	100.04 ± 10.08	97.75 ± 7.96	96.63 ± 6.97	0.06
Insulin (μUI/mL)	22.97 ± 10.87	19.76 ± 14.37	18.58 ± 12.24	0.11
HOMA-IR index	5.70 ± 2.87	4.83 ± 3.59	4.54 ± 3.27	0.10
Triglycerides (mg/dL)	134.79 ± 71.93	119.00 ± 55.46	112.25 ± 70.10	0.13
HDL-c (mg/dL)	42.81 ± 8.46*	46.72 ± 7.44*	44.91 ± 7.46	0.01

Values are mean ± SD; unless indicated.

*BMI* body mass index, *HOMA* homeostasis model assessment insulin resistance, *HDL-c* high-density lipoprotein cholesterol.

*P-values < 0.05 from post hoc analysis (Bonferroni).

^a^P-value obtained from one way ANOVA and χ^2^ test for quantitative and categorical variables, respectively.

^b^Socioeconomic status (SES) score was evaluated based on parental education level, parental job, family size, having car in the family, having computer/laptop, having personal room and having travel by using a validated questionnaire.

Age, sex and energy intake-adjusted dietary intakes of the study population across tertiles of legumes and nuts consumption are presented in Table 2. Persons in the top category of legumes and nuts compared to the bottom category had a significantly higher intake of protein, monounsaturated fatty acids (MUFA), vitamin A, vitamin C, riboflavin, folate, vitamin B12, magnesium, zinc, and dietary fiber. However, these participants had a significantly lower intake of carbohydrate, thiamin, niacin and vitamin B6 intake, compared to individuals in the first tertile.

**Table 2:** Dietary intakes (energy and macro/micro nutrients) of study participants across energy-adjusted tertiles of legumes and nuts consumption.

Energy-adjusted tertiles of legumes and nuts consumption				
	T1 (n = 67) (< 41.1 g/day)	T2 (n = 68) (41.1–71.1 g/day)	T3 (n = 68) (> 71.7 g/day)	P-value^a^
Energy, kcal	2972.11 ± 416.07	2818.93 ± 425.80	2859.33 ± 908.14	0.53
Protein, % of energy	13.21 ± 1.64	14.28 ± 1.54	15.42 ± 2.16	< 0.001
Carbohydrate, % of energy	60.00 ± 5.14	57.88 ± 4.77	57.03 ± 5.24	0.01
Fat, % of energy	27.93 ± 4.75	29.26 ± 5.04	29.34 ± 5.65	0.24
Cholesterol, mg	288.96 ± 138.36	268.75 ± 79.91	288.59 ± 129.24	0.54
SFA, g	27.28 ± 7.46	27.36 ± 6.95	27.42 ± 11.02	0.24
MUFA, g	26.85 ± 7.65	27.01 ± 7.78	28.78 ± 11.64	0.04
PUFA, g	29.15 ± 8.56	28.23 ± 7.82	28.07 ± 11.53	0.94
Vitamin C, mg	115.56 ± 65.43	131.42 ± 48.09	153.65 ± 75.24	< 0.001
Vitamin A, RAE	912.50 ± 603.66	1062.19 ± 567.59	1344.71 ± 781.99	< 0.001
Thiamin, mg	2.81 ± 0.42	2.57 ± 0.46	2.56 ± 0.81	0.01
Riboflavin, mg	2.14 ± 0.70	2.29 ± 0.55	2.46 ± 0.94	< 0.001
Niacin, mg	30.02 ± 5.83	26.57 ± 5.07	26.15 ± 9.63	< 0.001
Vitamin B6, mg	1.50 ± 0.50	1.58 ± 0.40	1.78 ± 0.73	< 0.001
Vitamin E, mg	32.64 ± 12.79	30.40 ± 11.14	28.06 ± 13.42	0.23
Folate, mcg	252.55 ± 81.15	303.21 ± 73.64	393.17 ± 140.81	< 0.001
Vitamin B12, mcg	4.03 ± 1.83	4.45 ± 1.39	4.81 ± 1.97	0.01
Magnesium, mg	257.45 ± 70.13	285.89 ± 48.86	320.77 ± 113.14	< 0.001
Zinc, mg	9.71 ± 2.58	10.64 ± 2.04	11.53 ± 4.24	< 0.001
Selenium, mcg	0.96 ± 0.03	0.95 ± 0.03	0.90 ± 0.04	0.43
Total fiber, g	17.09 ± 4.66	18.56 ± 4.04	22.66 ± 8.20	< 0.001

Values are mean ± SD. Energy intake and macronutrients were adjusted for age and gender; all other values were adjusted for age, gender and energy intake.

*SFA* saturated fatty acids, *MUFA* monounsaturated fatty acids, *PUFA* polyunsaturated fatty acids.

^a^P-value obtained from ANCOVA test for adjustment of energy intake.

**Figure 1:**
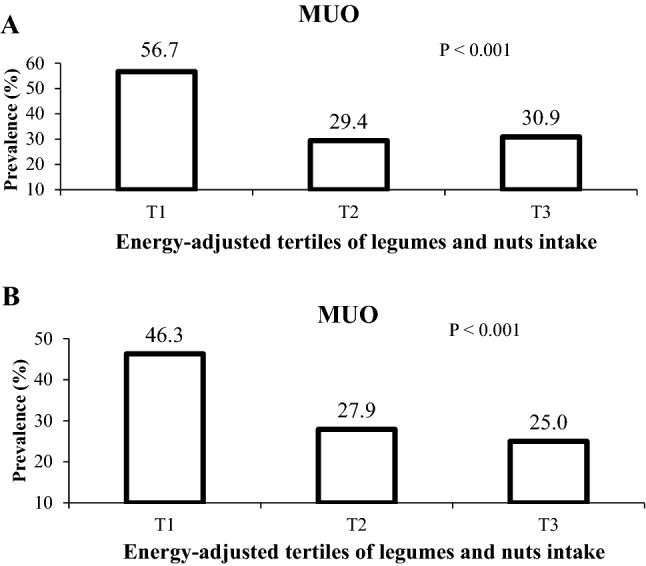
Prevalence of MUO ((**A**) based on both IDF and (**B**) IDF/HOMA-IR definition) in energy-adjusted tertiles of legumes and nuts consumption.

MUO prevalence based on IDF and IDF/HOMA-IR definitions in different energy-adjusted tertiles of legumes and nuts consumption is presented in Fig. 1**.** Participants in the highest tertile of legumes and nuts had lower prevalence of MUO based on both criteria.

Multivariate adjusted ORs and 95% CIs for MUO across energy-adjusted tertiles of legumes and nuts consumption are provided in Table 3**.** Based on IDF definition, in crude model, higher consumption of legumes and nuts was related to 66% decreased odds of MUO (OR = 0.34, 95% CI 0.17–0.69). After adjustment for potential confounders, the relation was not significant anymore (in fully-adjusted model: OR = 0.63, 95% CI 0.27–1.49). According to IDF/HOMA-IR definition, the same results were found; a 61% significant decrease in MUO odds was seen in crude model (OR = 0.39, 95% CI 0.19–0.80), but this relation was not significant in fully-adjusted model (OR = 0.69, 95% CI 0.28–1.70). Furthermore, in crude model, one tertile increment in legumes and nuts consumption was linearly related to 43% (OR = 0.57, 95% CI 0.40–0.82) and 39% (OR = 0.61, 95% CI 0.42–0.88) decreased odds of MUO, based on IDF and IDF/HOMA-IR definition, respectively. Again, these associations disappeared in fully adjusted model.

**Table 3:** Multivariate adjusted odds ratio (OR) and 95% confidence interval (CI) for MUO across energy-adjusted tertiles of legumes and nuts consumption^1^.

Energy-adjusted tertiles of legumes and nuts consumption					
	T1 (n = 67)	T2 (n = 68)	T3 (n = 68)	P-trend	Per 1 tertile increase
MUO phenotype based on IDF criteria					
Cases (n)	38	20	21		
Crude	1.00	0.32 (0.16, 0.65)	0.34 (0.17, 0.69)	0.01	0.57 (0.40, 0.82)
Model 1^a^	1.00	0.36 (0.17, 0.76)	0.39 (0.18, 0.82)	0.01	0.61 (0.41, 0.89)
Model 2^b^	1.00	0.35 (0.15, 0.81)	0.64 (0.27, 1.50)	0.23	0.77 (0.50, 1.18)
Model 3^c^	1.00	0.35 (0.15, 0.81)	0.63 (0.27, 1.49)	0.22	0.76 (0.46, 1.17)
MUO phenotype based on HOMA-IR criteria					
Cases (n)	31	19	17		
Crude	1.00	0.45 (0.22, 0.92)	0.39 (0.19, 0.80)	0.01	0.61 (0.42, 0.88)
Model 1^a^	1.00	0.52 (0.25, 1.11)	0.43 (0.20, 0.96)	0.40	0.65 (0.43, 0.97)
Model 2^b^	1.00	0.54 (0.23, 1.25)	0.72 (0.29, 1.74)	0.39	0.82 (0.52, 1.28)
Model 3^c^	1.00	0.56 (0.24, 1.29)	0.69 (0.28, 1.70)	0.35	0.80 (0.51, 1.26)

All values are odds ratios and 95% confidence intervals.

^a^Model 1: adjusted for age, sex, and energy intake.

^b^Model 2: additionally adjustment for physical activity and socioeconomic status (parental education, parental job, number of family members, having car in the family, having computer/laptop, having personal room and having trip).

^c^Model 3: additionally adjustment for body mass index (BMI).

Multivariate adjusted odds ratio (OR) and 95% confidence interval (CI) for MUO components across energy-adjusted tertiles of legumes and nuts consumption are reported in Table 4**.** In crude model, higher legume and nut consumption was significantly related to lower odds of hyperglycemia (OR = 0.29, 95% CI 0.14–0.60), low HDL-c (OR = 0.46, 95% CI 0.23–0.93) and insulin resistance (OR = 0.20, 95% CI 0.09–0.47). After adjustment for potential cofounders, only an inverse significant association was found between legumes and nuts consumption and odds of hyperglycemia (OR = 0.35, 95% CI 0.16–0.78).

**Table 4:** Multivariate adjusted odds ratio (OR) and 95% confidence interval (CI) for MUO components across energy-adjusted tertiles of legumes and nuts consumption.

	Energy-adjusted tertiles of legumes and nuts consumption			
	T1 (n = 67)	T2 (n = 68)	T3 (n = 68)	P-trend
Hyperglycemia (FBS ≥ 100)				
Cases (n)	38	27	19	
Crude	1.00	0.50 (0.25, 0.99)	0.29 (0.14, 0.60)	0.01
Fully adjusted model	1.00	0.50 (0.23, 1.6)	0.35 (0.16, 0.78)	0.01
Hypertriglyceridemia (TG ≥ 150)				
Cases (n)	23	14	15	
Crude	1.00	0.49 (0.22, 1.0)	0.54 (0.25, 1.16)	0.10
Fully adjusted model	1.00	0.52 (0.22, 1.20)	0.67 (0.28, 1.60)	0.31
Low HDL-c^a^				
Cases (n)	35	18	23	
Crude	1.00	0.32 (0.16, 0.6)	0.46 (0.23, 0.93)	0.02
Fully adjusted model	1.00	0.35 (0.15, 0.82)	1.07 (0.45, 2.55)	0.95
Hypertension (BP ≥ 130/85)				
Cases (n)	11	11	7	
Crude	1.00	0.98 (0.39, 2.44)	0.58 (0.21, 1.61)	0.31
Fully adjusted model	1.00	1.50 (0.56, 4.03)	0.67 (0.20, 2.18)	0.62
Insulin resistance (HOMA-IR score ≥ 3.16)				
Cases (n)	57	52	37	
Crude	1.00	0.57 (0.23, 1.36)	0.20 (0.09, 0.47)	< 0.001
Fully adjusted model	1.00	0.97 (0.31, 3.01)	0.41 (0.13, 1.24)	0.07

All values are odds ratios and 95% confidence intervals. Fully adjusted model: adjusted for age, sex, energy intake, physical activity, socioeconomic status (parental education, parental job, number of family members, having car in the family, having computer/laptop, having personal room and having trip) and body mass index (BMI).

^a^HDL-c < 40 mg/dL for the age of < 16 years, and < 50 mg/dL in girls/ < 40 mg/dL in boys for the age of ≥ 16 years).

The relation between energy-adjusted legumes and nuts consumption with odds of MUO, stratified by BMI status of participants, are presented in Table 5. In overweight participants, although there was an inverse significant association between intakes of legumes and nuts and odds of MUO in the crude model, this association disappeared after adjustment for all confounders. In obese subjects, there was no significant association in crude or adjusted models, based on both definition criteria.

**Table 5:** Multivariate adjusted odds ratio (OR) and 95% confidence interval (CI) for MUO across energy-adjusted tertiles of legumes and nuts consumption stratified by BMI categories.

Energy-adjusted tertiles of legumes and nuts consumption				
	T1	T2	T3	P-trend
MUO phenotype based on IDF criteria				
Overweight (cases/participants)	18/32	3/35	7/37	–
Crude	1.00	0.73 (0.02, 0.29)	0.18 (0.06, 0.53)	0.01
Model 1^a^	1.00	0.08 (0.02, 0.34)	0.21 (0.07, 0.69)	0.01
Model 2^b^	1.00	0.05 (0.01, 0.28)	0.73 (0.16, 3.30)	0.18
Obese (cases/participants)	20/35	17/33	14/31	–
Crude	1.00	0.80 (0.31, 2.07)	0.62 (0.34, 1.64)	0.33
Model 1^a^	1.00	0.94 (0.34, 2.58)	0.65 (0.23, 1.82)	0.42
Model 2^b^	1.00	1.02 (0.34, 3.08)	0.69 (0.22, 2.14)	0.53
MUO phenotype based on IDF /HOMA-IR criteria				
Overweight (cases/participants)	12/32	3/35	5/37	–
Crude	1.00	0.16 (0.04, 0.62)	0.26 (0.08, 0.85)	0.02
Model 1^a^	1.00	0.19 (0.05, 0.79)	0.38 (0.11, 1.34)	0.09
Model 2^b^	1.00	0.11 (0.02, 0.68)	2.10 (0.38, 11.49)	0.90
Obese (cases/participants)	19/35	16/33	12/31	–
Crude	1.00	0.79 (0.31, 2.06)	0.53 (0.20, 1.42)	0.21
Model 1^a^	1.00	0.93 (0.34, 2.55)	0.54 (0.19, 1.53)	0.25
Model 2^b^	1.00	1.01 (0.34, 3.01)	0.54 (0.17, 1.69)	0.31

All values are odds ratios and 95% confidence intervals.

^a^Model 1: adjusted for age, sex and energy intake.

^b^Model 2: additionally adjustments for physical activity and socioeconomic status (parental education, parental job, number of family members, having car in the family, having computer/laptop, having personal room and having trip).

The associations between energy-adjusted legumes and nuts consumption with odds of MUO, stratified by sex of participants, are provided in Table 6**.** In girls, there was an inverse significant association between consumption of legumes and nuts and odds of MUO based on both criteria in the crude model. But after adjustment for physical activity, socioeconomic status and BMI, this relation was not significant. In boys, there was no significant association between legumes and nuts consumption and MUO, based on both definitions, in the crude or adjusted models.

**Table 6:** Multivariate adjusted odds ratio (OR) and 95% confidence interval (CI) for MUO across energy-adjusted tertiles of legumes and nuts consumption stratified by sex.

Energy-adjusted tertiles of legumes and nuts consumption				
	T1	T2	T3	P-trend
MUO phenotype based on IDF criteria				
Girls (cases/participants)	22/33	10/34	10/35	–
Crude	1.00	0.21 (0.07, 0.59)	0.20 (0.07, 0.56)	0.01
Model 1^a^	1.00	0.22 (0.07, 0.67)	0.27 (0.09, 0.79)	0.02
Model 2^b^	1.00	0.26 (0.81, 0.87)	0.51 (0.15, 1.77)	0.22
Model 3^c^	1.00	0.26 (0.77, 0.85)	0.52 (0.15, 1.82)	0.23
Boys (cases/participants)	16/34	10/34	11/33	–
Crude	1.00	0.47 (0.17, 1.27)	0.56 (0.21, 1.51)	0.24
Model 1^a^	1.00	0.55 (0.19, 1.54)	0.57 (0.19, 1.70)	0.30
Model 2^b^	1.00	0.37 (0.11, 1.29)	0.74 (0.20, 2.67)	0.57
Model 3^c^	1.00	0.39 (0.11, 1.35)	0.64 (0.17, 2.40)	0.42
MUO phenotype based on IDF /HOMA-IR criteria				
Girls (cases/participants)	16/33	10/34	6/35	–
Crude	1.00	0.44 (0.16, 1.21)	0.22 (0.07, 0.67)	0.01
Model 1^a^	1.00	0.47 (0.16, 1.41)	0.29 (0.09, 0.97)	0.04
Model 2^b^	1.00	0.67 (0.20, 2.21)	0.56 (0.14, 2.14)	0.37
Model 3^c^	1.00	0.67 (0.20, 2.23)	0.55 (0.14, 2.13)	0.37
Boys (cases/participants)	15/34	9/34	11/33	–
Crude	1.00	0.44 (0.16, 1.26)	0.63(0.23, 1.70)	0.35
Model 1^a^	1.00	0.56 (0.19, 1.63)	0.70 (0.23, 2.09)	0.50
Model 2^b^	1.00	0.39 (0.11, 1.37)	0.96 (0.26, 3.53)	0.85
Model 3^c^	1.00	0.40 (0.11, 1.41)	0.86 (0.23, 3.27)	0.70

All values are odds ratios and 95% confidence intervals.

^a^Model 1: adjusted for age, sex and energy intake.

^b^Model 2: additionally adjustments for physical activity and socioeconomic status (parental education, parental job, number of family members, having car in the family, having computer/laptop, having personal room and having trip).

^c^Model 3: additionally adjustment for body mass index (BMI).

## Discussion

In this study, overweight/obese adolescents with higher legumes and nuts consumption compared to those with lower intake had lower odds of MUO based on IDF, IDF/HOMA-IR in the crude model and after considering main confounders in model 1. But after further adjustments for physical activity, socioeconomic status, and BMI, there was no significant association. Moreover, in girls and overweight participants, we found an inverse significant association, based on both definition criteria in crude model; however, these associations disappeared after considering all potential confounders. There was no significant association in boys or obese subjects, before or after taking potential confounders into account.

Regarding the increasing rate of childhood overweight and obesity, it must be considered that MHO is not a constant condition and children may be transferred from MHO to MUO, as they get older^1,13^. Considering the high morbidity and mortality due to childhood obesity^33^, it is worthy to prevent the incidence of these disorders and their consequences. In the current study, we illustrated that higher intakes of legumes and nuts might be related to decreased odds of MUO. Therefore, it could be clinically advised to adolescents to pay more attention to consumption of legumes and nuts for preventing MUO and its adverse effects.

Similar to the current study, some previous cross-sectional studies have examined the relationship between legumes and nuts consumption and metabolic risk factors in adolescents. Following a Mediterranean diet was related to a lower risk of MUO in European overweight or obese adolescents^34^. Mirmiran et al. have reported a non-significant decrease in metabolic syndrome (MetS) prevalence and its components across the quartiles of nuts and dried fruit consumption in adolescents. But they found a significant decreasing trend in TG concentration across quartiles of nuts and dried fruit consumption^35^. Moreover, Aghayan et al. illustrated an inverse association between nut consumption and risk of carotid intima-media thickness (cIMT) in overweight and obese pediatrics^36^. In addition, higher soy intake was inversely associated with the prevalence of hypertension and obesity in Chinese children and adolescents^37^. It is worth noting that genetic, dietary habits and life style are critical factors that influence incidence of metabolic disorders; these factors vary in different societies. Therefore, it is necessary to find the main risk factors for prevention of metabolic disorders in each nation. In the current analysis, we investigated Iranian overweight and obese adolescent and illustrated that higher habitual intake of legumes and nuts was linked to lower likelihood of MUO in crude model. Nevertheless, this association disappeared after considering socio-economic status, physical activity and weight status of subjects. In other words, adolescents with higher consumption of nuts and legumes might have better socio-economic status, healthier life style and normal weight. Therefore, a combination of these variables with consumption of nuts and legumes resulted in healthier metabolic health status in Iranian adolescents.

Epidemiologic studies on adults have also demonstrated an inverse association between regular legume intake and MetS and its components^38,39^. Additionally, a randomized controlled trial in adults with MetS illustrated that consumption of 30 gr/day soy protein could decrease BMI, weight, total cholesterol, LDL-c, and Apo B and increased HDL-c^40^. Another clinical trial revealed that adherence to the Mediterranean diet for 12 weeks had a favorable effect on oxidative stress, inflammation, liver hepatic steatosis, insulin resistance, BMI and body fat in adolescents with non-alcoholic fatty liver disease^41^. The contradictories in findings of previous investigations might be due to variations in study designs, study subjects, confounders, and multiple methods applied to assess dietary intakes.

Prior studies suggested some pathways to explain the positive effect of legumes and nuts on cardiometabolic risk factors. Legumes, soy and nuts are rich sources of protein, fiber, polyphenols, potassium, and magnesium^42^. Plant proteins have a high value of glutamic acid, a precursor for arginine. Therefore, legumes and nuts could decrease blood pressure through the production of nitric oxide^43^. Also, considering the role of potassium in insulin secretion, potassium intake from legumes and nuts have a favorable effect on glucose intolerance^44^. In addition, soluble fibers can decrease the absorption rate of nutrients and increase the excretion of bile acid and cholesterol; these fibers have also a positive effect on gut microbiota, hepatic gluconeogenesis, lipogenesis, and lipid storage through the synthesis of short-chain fatty acids (SCFAs). Moreover, the high fiber content of legumes and nuts can lead to low energy density of foods and consequently a decrease in risk of obesity and its complication^45^.

The current analysis investigated the relation of legumes and nuts consumption with MUO in Iranian overweight and obese adolescents who had different dietary intakes and cultural features from other societies. More than 55% of total energy intake among Iranian population is derived from carbohydrates, especially refined grains^46^. Moreover, two different definitions were used to categorize participants as MHO and MUO. Furthermore, several potential confounders were controlled in the analyses. However, several restrictions should be considered. Considering the cross-sectional design of the current study, it was impossible to define the causality of the relation of legumes and nuts consumption with MUO. Therefore, further prospective studies are required to confirm the causality of this relation. Although dietary intake assessment was done through a valid FFQ, some unavoidable biases such as misclassification and recall bias might have influenced our findings. We made adjustments for several confounders; however, the probable effects of residual confounders (birth weight, sleeping deprivation, maturity of persons, food habits, and BMI of parents) were not controlled. Overweight or obesity was defined through BMI; nevertheless, body composition and fat distribution, two critical factors that could affect metabolic health status, were not measured.

In conclusion, after taking potential confounders into account, no significant association was found between consumption of legumes and nuts and MUO in Iranian adolescents. The findings should be affirmed by further prospective studies.

## Data Availability

The data that support the findings of this study are available from the corresponding author upon reasonable request.

## Abbreviations

GBD: Global burden of disease
BP: Blood pressure
MHO: Metabolically healthy overweight or obese
MUO: Metabolically unhealthy overweight or obese
DASH: Dietary approaches to stop hypertension
BMI: Body mass index
TC: Total cholesterol
TG: Triglycerides
HDL-c: High density lipoprotein cholesterol
LDL-c: Low density lipoprotein cholesterol
SBP: Systolic blood pressure
CI: Confidence interval
FFQ: Food frequency questionnaire
WHO: World Health Organization
DBP: Diastolic blood pressure
FBG: Fasting blood glucose
IR: Insulin resistance
HOMA-IR: Homeostasis model assessment insulin resistance
IDF: International Diabetes Federation
SES: Socioeconomic status
ANOVA: One-way analysis of variance
ANCOVA: Analysis of covariance
SPSS: Statistical package for the social sciences
SD: Standard deviation
SE: Standard error
cIMT: Carotid intima-media thickness
SCFAs: Short-chain fatty acids
PAQ-A: Physical Activity Questionnaire for Adolescents
MetS: Metabolic syndrome
